# Genome-wide identification and expression analysis of the U-box E3 ubiquitin ligase gene family related to salt tolerance in sorghum (*Sorghum bicolor* L.)

**DOI:** 10.3389/fpls.2023.1141617

**Published:** 2023-03-17

**Authors:** Jianghui Cui, Genzeng Ren, Yuzhe Bai, Yukun Gao, Puyuan Yang, Jinhua Chang

**Affiliations:** ^1^ College of Agronomy, Hebei Agricultural University, Baoding, China; ^2^ North China Key Laboratory for Germplasm Resources of Education Ministry, Baoding, China

**Keywords:** ubiquitin ligases, U-box, sorghum, salt tolerance, gene family

## Abstract

Plant U-box (PUB) E3 ubiquitin ligases play essential roles in many biological processes and stress responses, but little is known about their functions in sorghum (*Sorghum bicolor* L.). In the present study, 59 *SbPUB* genes were identified in the sorghum genome. Based on the phylogenetic analysis, the 59 *SbPUB* genes were clustered into five groups, which were also supported by the conserved motifs and structures of these genes. *SbPUB* genes were found to be unevenly distributed on the 10 chromosomes of sorghum. Most *PUB* genes (16) were found on chromosome 4, but there were no *PUB* genes on chromosome 5. Analysis of *cis*-acting elements showed that *SbPUB* genes were involved in many important biological processes, particularly in response to salt stress. From proteomic and transcriptomic data, we found that several *SbPUB* genes had diverse expressions under different salt treatments. To verify the expression of *SbPUBs*, qRT-PCR analyses also were conducted under salt stress, and the result was consistent with the expression analysis. Furthermore, 12 *SbPUB* genes were found to contain MYB-related elements, which are important regulators of flavonoid biosynthesis. These results, which were consistent with our previous multi-omics analysis of sorghum salt stress, laid a solid foundation for further mechanistic study of salt tolerance in sorghum. Our study showed that *PUB* genes play a crucial role in regulating salt stress, and might serve as promising targets for the breeding of salt-tolerant sorghum in the future.

## Introduction

The ubiquitin/26S proteasome (UPS) pathway, which degrades ubiquitinated target proteins, mainly includes the E1 ubiquitin (UB) activating enzyme, E2 UB binding enzyme, and E3 UB ligase; these proteins are involved in a wide variety of cellular processes (Vierstra, 2003; Vierstra, 2009). UPS regulates a diverse array of plant growth and development processes, as well as the degradation of short-lived proteins (Wang and Deng, 2011). E3 ligases are considered to be necessary for ubiquitin activation and transfer, and play a key role in protein ubiquitination(Yee and Goring, 2009; Trujillo, 2018). Based on the mechanism of action and the presence of conserved domains, E3 ubiquitin ligases can be classified into two main types, multi-subunit types and single subunit types, which are composed of Homologous to E6-associated protein Carboxyl Terminus (HECT), Really Interesting New Gene (RING) finger and U-box domains (Buetow and Huang, 2016; Yang et al., 2021). The ubiquitin ligases promote the covalent binding of ubiquitin to target proteins in eukaryotes, resulting in the addition of a single protein or protein complex to the ubiquitin reaction (Berndsen and Wolberger, 2014; Wang et al., 2022). The U-box domain consists of about 75 amino acids, and many plant U-box (PUB) proteins play a crucial role in the development and physiological processes, such as plant hormone responses, seed germination, and responses to biotic and abiotic stresses (Ohi et al., 2003; Zeng et al., 2006; Mao et al., 2022).

Environmental stress has adverse effects on plant growth and development (Cramer et al., 2011). Excessive salinity in the soil had a harmful impact on plant growth and productivity leading to large reductions in grain yield (Shrivastava and Kumar, 2015; Dadrwal et al., 2020). Under salt stress, a large number of signal pathways in higher plants may be activated to regulate the expression of different types of genes and to produce a variety of defense proteins and protective molecules (Zhu, 2002; Shomali and Aliniaeifard, 2020). Therefore, the identification and functional analysis of salt stress-related genes are important for crop improvement. At present, many PUB proteins are associated with salt tolerance in several important crops. For example, AtPUB18, AtPUB19, and AtPUB44 have been found to have negative regulatory effects on seed germination under salt stress and abscisic acid (ABA) treatment in *Arabidopsis* (Bergler and Hoth, 2011; Salt et al., 2011). Overexpression of *AtPUB22* and *AtPUB23* resulted in hypersensitivity to salt stress and drought stress; further, mutants of these two genes had enhanced drought tolerance, indicating that these PUB proteins play a negative regulatory role in response to abiotic stresses (Cho et al., 2008). *OsPUB15* mutants in rice had defects in seed growth, but overexpression of *OsPUB15* produced plants with higher salt tolerance than the wild type (Park et al., 2011). Wheat *TaPUB26* was shown to negatively regulate salt stress in transgenic *Brachypodium distachyon*. Additionally, overexpression of *TaPUB26* enhanced the accumulation of reactive oxygen species and could affect the cytoplasmic Na^+^/K ^+^ balance (Wu et al., 2020). *TaPUB1* positively regulated salt stress tolerance in wheat and tobacco. Overexpression of *TaPUB1* up-regulated the expression of genes related to ion channels, increasing net root Na^+^ efflux but decreasing net K^+^ efflux and H^+^ influx, thus maintaining a lower ratio of Na^+^/K^+^ cytosolic solute (Zhang et al., 2017b; Wang et al., 2020). During seed germination and post-germination growth stages, overexpression of the *GmPUB8* gene led to the hypersensitivity of soybean to salt and drought stress (Wang et al., 2016). In the Antarctic moss *Pohlia nutans*, the growth of gametophyte was more sensitive to salinity and ABA with heterogeneous overexpression of *PnSAG1*, which was thought to play a negative role in plant responses to ABA and salt stress (Wang et al., 2019).

Sorghum (*Sorghum bicolor* L.) is the fifth most important crop in the world; it originated in Africa and is the most widely utilized C4 model crop (Mullet et al., 2014; Enyew et al., 2022; Espitia-Hernández et al., 2022). In addition, sorghum is one of the most important food crops worldwide, especially in developing countries and areas with more drought and salinity (Zhao et al., 2019; Tasie and Gebreyes, 2020). Compared with other cereals gains, sorghum can better adapt to a variety of environments and can be cultivated under more adverse conditions (Borrell et al., 2006). Sorghum is a model system for studying crop salt response because of its inherent tolerance to salt stress. Based on the analysis of multi-omics data, it was determined that flavonoid biological pathways play a vital role in the salt tolerance of sorghum (Ren et al., 2022). Moreover, ubiquitin-mediated proteolysis was found among the top Kyoto Encyclopedia of Genes and Genomes (KEGG) pathways of sorghum salt tolerance. To date, PUB proteins are related to salt tolerance in many plants, but have been rarely studied in sorghum (Wang et al., 2016; Trujillo, 2018; Mao et al., 2022). In this study, we identified *PUB* genes genome-wide and analyzed their gene structure and evolution. Using transcriptomic and proteomic data for sorghum, we demonstrated that several *SbPUB*s showed different expression responses to salt stress. In addition, we found that the MYB-related elements, which are important regulators of flavonoid biosynthesis (Falcone Ferreyra et al., 2012; Zhai et al., 2016), were widely distributed in the *SbPUB*s. This study provides a theoretical basis for analyzing the role of *PUB* genes in the salt tolerance of sorghum.

## Materials and methods

### Genome identification of U-box gene family members in sorghum

In order to identify potential members of the *PUB* gene family, the genome sequences of sorghum (*Sorghum_bicolor*_NCBIv3) were first downloaded from the Ensembl Plants database (Yates et al., 2022). Then, the seed file of the U-box domain (PF04564) was used to search the candidate *PUB* genes in the sorghum protein database using the software HMMER (v3.3.2) (Mistry et al., 2013). All candidate SbPUB proteins, which were obtained from the result of HMM search, were further submitted to the SMART website (http://smart.embl-heidelberg.de/) to determine the completeness of the U-box conserved domain. The tool Compute pI/Mw on the ExPasy website (https://web.expasy.org/compute_pi/) was used to obtain the molecular weight (MW) and isoelectric point (pI) (Gasteiger, 2005).

### Phylogenetic analysis of *SbPUB* proteins

The software ClustalX (v2.1) was used to perform multiple sequence alignment (Larkin et al., 2007). Subsequently, the phylogenetic tree was constructed by the MEGA (v7.0) tool using the Neighbor-joining method with a bootstrap of 1000 replications (Kumar et al., 2018). The Neighbor-Joining (NJ) method, which uses an agglomerative process and is both fast and accurate, is the most commonly used distance-based method for phylogenetic analysis (Saitou and Nei, 1987). The alignments were edited by the GeneDoc (v2.7) sequence editor (Nicholas and Nicholas, 1996). The Evolview tool (https://evolgenius.info//evolview-v2/) was used to edit the phylogenetic tree of SbPUB proteins (Zhang et al., 2012). To explore the diversity, PUB proteins of rice and *Arabidopsis thaliana* were retrieved from previous studies in the National Center for Biotechnology Information (NCBI) database (Wiborg et al., 2008; Zeng et al., 2008).

### Structural characteristics, motif, and *cis*-acting elements analysis

To detect the conserved motifs of SbPUB proteins, Multiple Em for Motif Elicitation (MEME v5.2.0) (Bailey et al., 2009) was used with the maximum number of motifs set as 10 (Bailey et al., 2009). And the intron-exon gene structures were displayed using TBtools with the *gff3* files of the sorghum genome (Chen et al., 2020). The upstream 2 kb region of each *SbPUB* gene was defined as a putative promoter sequence, which was extracted and submitted to the PlantCARE database to predict the *cis*-regulatory elements with the default parameters (Lescot et al., 2002).

### Chromosomal and subcellular location, synteny analysis, and selective pressure estimation

The homologous gene pairs of *SbPUB*s were identified by blast software with an all-*vs*-all blast strategy. Then, the synteny regions were identified using MCScanX with the result of the all-vs-all blast (Wang et al., 2012). We plotted the circos picture to show the distribution of synteny gens pairs (Krzywinski et al., 2009). In order to predict the protein-protein interactions (PPIs), the STRING database was used for the candidate proteins (Szklarczyk et al., 2021). We utilized the Protein Data Bank and SWISS-MODEL to investigate the three-dimensional (3D) structures of the target proteins *via* comparative modeling (Waterhouse et al., 2018; Bittrich et al., 2022). Additionally, we employed PyMOL to visualize the protein 3D structures (DeLano, 2004). The chromosomal location analysis was conducted by Mapchart (v2.32) (Voorrips, 2002). The subcellular localization of sorghum U-box protein was predicted using the online software WolfPSORT (https://wolfpsort.hgc.jp/).

### Expression analysis of *SbPUB* genes

To study the expression patterns of *SbPUB* genes, proteomic data of two sorghum cultivars Henong16 (HN) and Gaoliangzhe (GZ) under different salt treatments time (0h, 24h, 48h, and 72h) were obtained through the ProteomeXchange Consortium (http://proteomecentral.proteomexchange.org) (Accession number PXD032125). Hierarchical clustering of the expression profiles was performed on the log-transformed fold change expression values for the identified protein spots using Proteome Discoverer 1.4 software as previously described (Orsburn, 2021). In addition, the RNA-seq data generated and analyzed in a previous study were used for this study (Accession Number: GSE145748) (Sun et al., 2020). The abundance of transcriptional data was expressed as fragments per kilobase of exon per million fragments mapped (FPKM). The heatmap of *SbPUB* genes was constructed by the package ggplot2 in R.

### Plant materials and treatments

The sorghum seeds of salt-sensitive HN16 and salt-tolerant GZ genotypes were selected for this study. The seeds were disinfected with 75% ethanol and then rinsed clean with deionized water. Subsequently, the sowing seeds were made in plastic containers with vermiculite. Three-day-old seedlings were transferred to hydroponic boxes and grown in an artificial climate chamber. When sorghum seedlings grow to three-leaf stages (about 15 days), they are transferred to a salt environment (0.6% NaCl) or the nutrient solution (control condition). Whole plant tissue samples were collected at 0, 24, 48, and 72 h of treatment, snap-frozen in liquid nitrogen, and stored at -80°C until RNA extraction. Experimental repeat runs for three biological and three technical replicates were included in the analysis.

### RNA isolation, reverser transcription, and quantitative real-time PCR analysis

Total RNAs were extracted from the salt stress-treated samples using the Omini Plant RNA Kit (CWBIO, Beijing, China). RNA was reverse transcribed into cDNA using a SuperRT cDNA Synthesis Kit (CWBIO, Beijing, China). Then, the cDNA was diluted 1:20 with ddH2O to be used as a template for quantitative real-time PCR (qRT-PCR). Then, qRT-PCR was performed using an AugeGreen™ qPCR Master Mix (US EVERBRIGHT, Suzhou, China) and samples were run in a LightCycler 96 System (Roche, CA, United States) using the following protocol: predenaturation at 95°C for 2 min; 45 cycles of denaturation at 95°C for 15 s and renaturation at 60°C for 60 s; and extension at 72°C for 30 s. The β-actin gene (X79378) was used as an internal reference gene. The primer pairs for qRT-PCR were summarized in **Table S5**. The relative expression levels were calculated using the 2^-ΔΔCt^ method, and three biological replicates and three technical replicates were performed for each sample.

## Results

### Identification of *SbPUB* gene family members

In this study, 59 *PUB* genes were identified in the entire sorghum genome and then were designated as *SbPUB* 1-59 based on their chromosomal location. To characterize these *SbPUB* genes, the length of the open reading frame and the protein sequence, theoretical isoelectric point (pI), protein molecular weight (MW), and subcellular and chromosomal locations were analyzed (**Table S1**). The length of *SbPUB*s ranged from 1,068 bp (*SbPUB43*) to 29,965 bp (*SbPUB57*), and the average length was 4,224 bp. Among the 59 *SbPUB* genes, the longest sorghum U-box protein was SbPUB13, containing 1,404 amino acids, and the shortest (SbPUB42) contained 275 amino acids. The MW ranged from 30.87 kDa to 150.37 kDa, with an average of 69.89 kDa. The pI value ranged from 4.99 (SbPUB2) to 8.82 (SbPUB55). The subcellular locations of SbPUBs were predicted by WolfPSORT; we found that most PUB proteins (56) were located on the endoplasmic reticulum (21), nucleus (19), and plasma membrane (16), except for two located in mitochondria and one located in the cytoplasm (**Table S1**). Among *SbPUB*s, the transcription direction of 29 genes was the same as the sorghum genome sequence, and the other 30 were in the opposite direction.

### Phylogenetic analysis and classification of *SbPUB* gene family members

**Figure 1:**
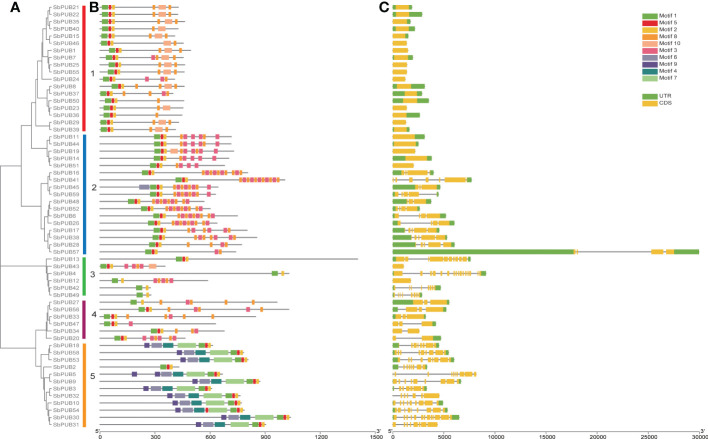
The phylogenetics, conserved motifs, and gene structure analysis of *SbPUB* proteins. **(A)** The Neighbor-Joining (NJ) phylogenetic tree of 59 sorghum *PUB* genes. Different colors of the branches represent five groups with marking in Latin letters. **(B)** The conserved motifs analysis of *PUB* genes in sorghum. Ten different motifs were predicted by MEME software, with the scale bar at the bottom. **(C)** The gene structure analysis of *SbPUB* genes using the tool of GSDS, including intron (grey lines), UTR (green rectangles), and exon (orange rectangles).

To investigate the evolutionary history of *PUB* genes in sorghum, we constructed a phylogenetic tree using MEGA (v7.0) with the neighbor-joining (NJ) method. The topology of the phylogenetic tree could be divided into five groups (**Figure 1A**). Group I was the largest with 18 genes, followed by Groups II and V with 17 genes and 12 genes, respectively, while Groups III and IV were the smallest with six genes each (**Table S1**).

To further investigate the relationships of the *SbPUB* genes in the phylogenetic tree, the conserved motifs were evaluated using the program MEME. Ten motifs were identified and estimated in the 59 *SbPUB* genes. Among them, Motif 1, Motif 2, Motif 5, and Motif 8 were found in most genes of the five groups, indicating that they are highly conserved in SbPUB proteins (**Figure 1B**)., Motif 1, Motif 2, and Motif 5 were determined to be conserved U-box sequences, which could be necessary to maintain the U-box structure and support their ubiquitin linkage activity. Motif 8 was part of the ARM conserved domain, which was the most common type in the U-box family. Furthermore, the genes in the same group within the phylogenetic tree exhibited similar conserved motifs, which indicated that they might have similar functions. For example, nearly all genes (10 out of 11) of Group V had 7 motifs (Motifs 1, 2, 4, 5, 6, 7, and 9).

To better understand the composition and function of the *SbPUB* genes, gene structure analysis was performed with the conserved sequences and exon/intron positions. We found that the number of exons varied from 1 to 18 in *SbPUB*s, which indicates that the sorghum *PUB* genes have complex RNA splicing processes. Genes in the same group had similar exon/intron structures (**Figure 1C**). The 18 genes in Group I all contained one to three exons, with an average of two, which was the least among the five groups. Group V had the most exons with an average of ten exons. The gene structure and conserved motif analyses were consistent with the *SbPUB* phylogenetic tree, which provided strong evidence for the accuracy of the classifications in the phylogenetic tree.

**Figure 2:**
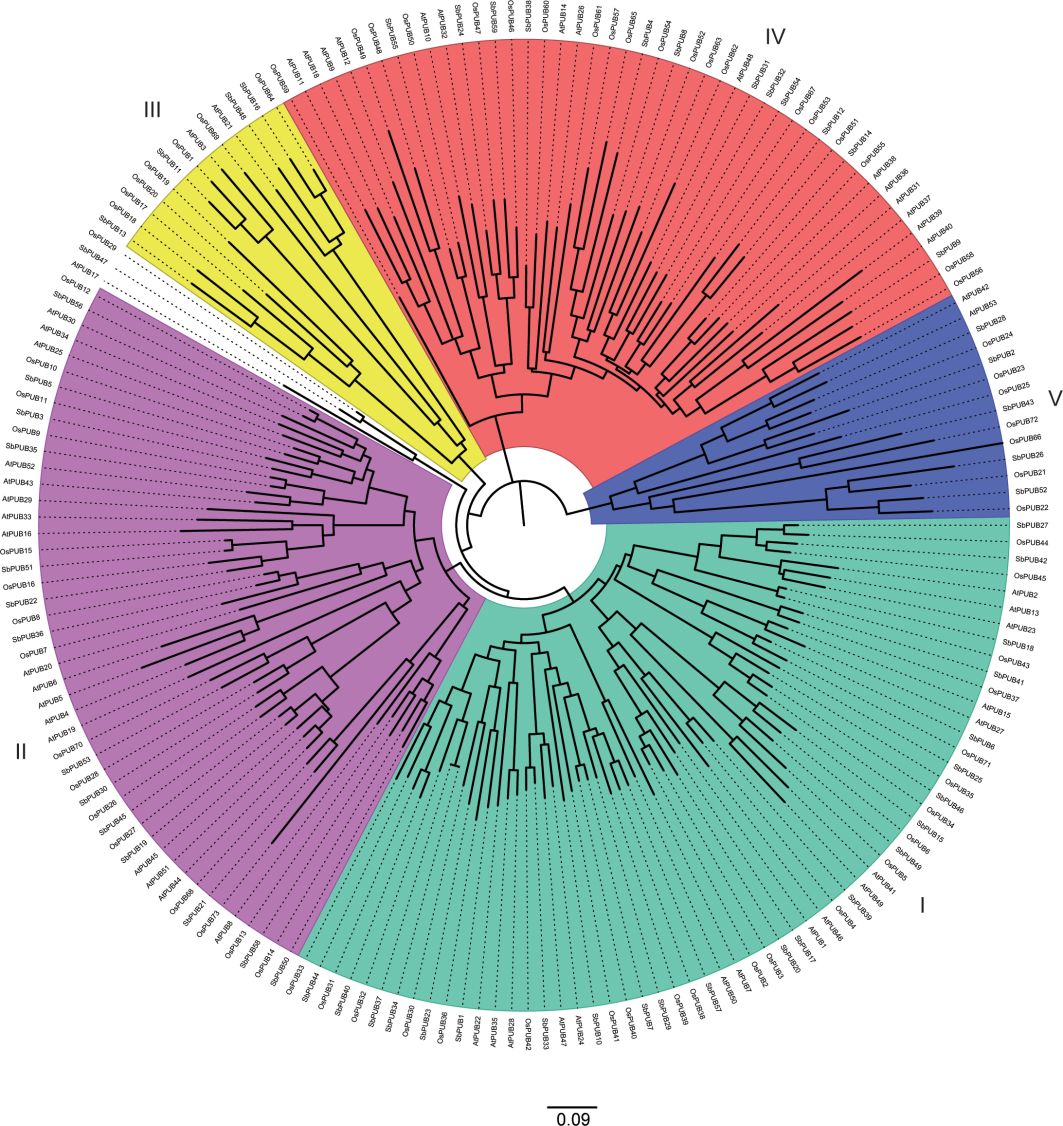
Phylogenetic tree analysis of the *PUB* gene family of sorghum, rice, and *Arabidopsis*. The multiple sequence alignment of the full-length sequences of these *PUB* proteins was performed by CluastalX, and the phylogenetic tree was constructed using the software MEGA 7 with the NJ method. All 185 PUB proteins were clustered into five subgroups, designated as Group I -V clades with different colors marked.

In addition, to further investigate the evolution of the *PUB* family, 185 PUB proteins (59 from sorghum, 73 from rice, and 53 from *A. thaliana*) (**Table S2**) were used to generate a phylogenetic tree (**Figure 2**). Based on the resulting phylogenetic tree, these PUB proteins could be divided into five subgroups. Group I contained the largest number of *PUB* genes of 62, while Group III had the least number of *PUB* genes of 12. In general, the *PUB* genes of sorghum and rice were more closely related to each other than those of *A. thaliana*, which is consistent with previous results. These results further support the accuracy of the *SbPUB* phylogenetic analysis in this study.

### Chromosomal location and homologous gene analysis of *SbPUB* genes

**Figure 3:**
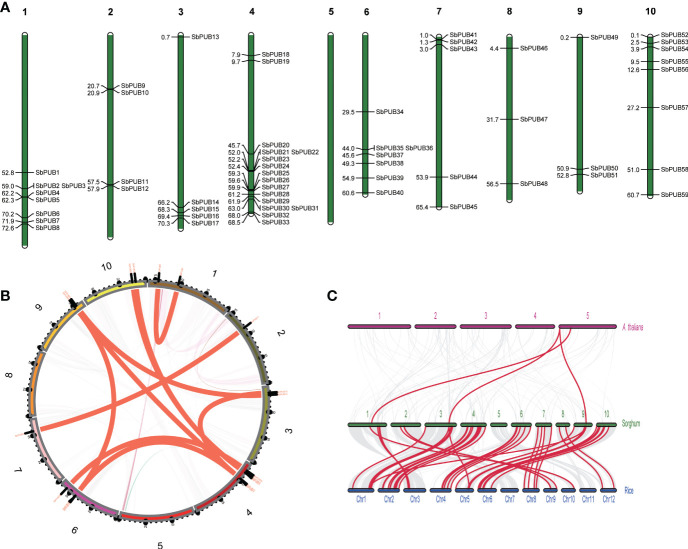
The location and synteny of *SbPUB* genes in the sorghum genome. **(A)** The distribution pattern of *PUBs* in 10 sorghum chromosomes. **(B)** The distribution pattern synteny analysis of *SbPUB* genes. The red lines represent the synteny gene pairs of *PUB* gene families in sorghum. **(C)** Collinearity analysis between sorghum, rice, and *A thaliana*. The gray lines represent the co-collinearity of all genes between different species, and the red lines represent the collinearity between different members of the *PUB* gene family.

The chromosomal distribution of *SbPUB*s in the genome was identified by extracting chromosomal data and mapping with MapChart (Voorrips, 2002). As a result, 59 *SbPUB* genes were mapped unevenly and nonrandomly onto 9 chromosomes, and no *SbPUB* gene was mapped onto chromosome 5 (**Figure 3A**). Chromosome 4 had the most *SbPUB* genes of 16, followed by chromosomes 1 and 10 with 8 genes. Each of chromosomes 3, 6, and 7 contained 5 to 7 *SbPUB* genes. Three or four *PUB* genes were mapped onto chromosomes 2, 8, and 9. Gene duplication events are significant for gene family expansion. Here, the duplication events of the *PUB* genes in the sorghum genome were analyzed using MCScanX software. We found that 30 genes were derived from dispersed duplication blocks: 31 genes were assigned to whole-genome duplication (WGD) and segmental duplication. In addition, there were five genes belonging to tandem duplication blocks, and 2 genes were derived from proximal duplication, while only one gene was assigned to the singleton category. Therefore, the dispersed, WGD and segmental duplication events (approximately 86.5%) may have resulted in the expansions of the *PUB* gene family in sorghum. It is thought that most duplicated genes will be silenced over time, but there are still a few maintained by purifying selection. A total of 11 homologous gene pairs were identified in the sorghum *PUB* gene family, which contained 18 homologous genes. Two homologous gene pairs were detected between chromosomes 4 and chromosomes 10 (**Figure 3B**). Further, the collinear relationship among sorghum, rice, and *A. thaliana* was identified (**Figure 3C**). There were 43 orthologous gene pairs between *SbPUB*s and *OsPUB*s, of which 3 *OsPUB*s had two orthologous copies in sorghum. These findings suggest that these orthologous genes might share similar functions in these species. In addition, we found four orthologous gene pairs between *SbPUB*s and *AtPUB*s, which was far less than those found for sorghum and rice, suggesting that *SbPUB* genes have a closer relationship with *OsPUB*s.

### C*is*-acting elements prediction of *SbPUB* genes

**Figure 4:**
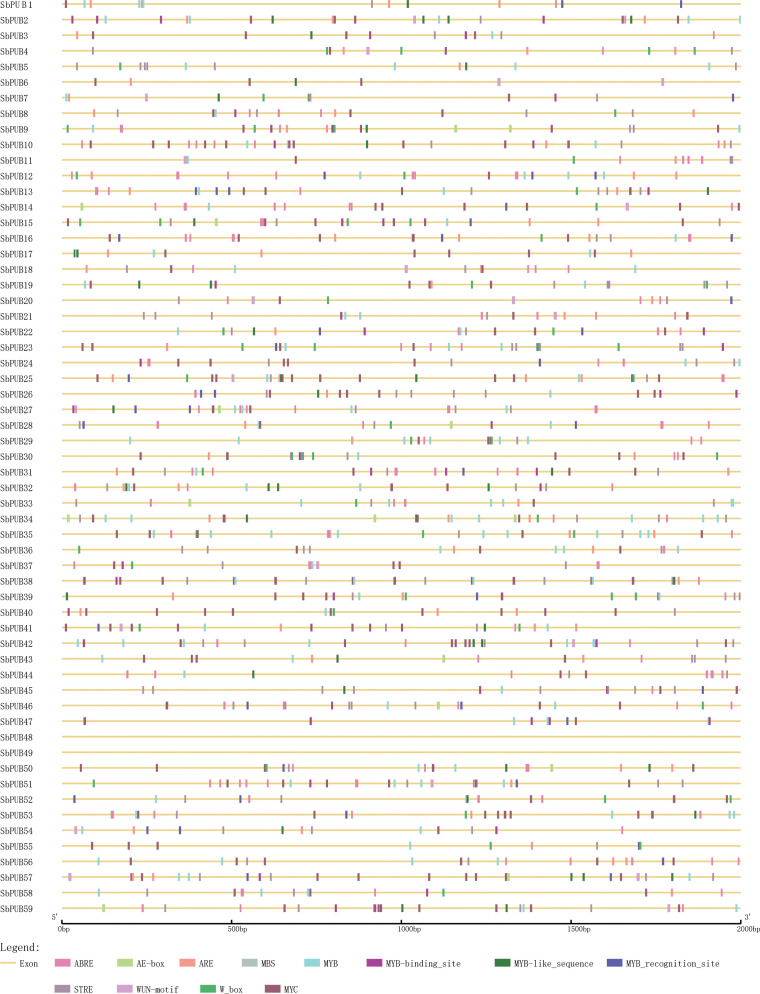
The *cis*-acting elements analysis of the putative promoter of 59 *SbPUB* genes. The distribution pattern of 12 elements of the putative promoter of the *PUB* gene family in sorghum, which function was related to salt stress. The color scale at the bottom represents these cis-acting elements, especially MYB-related elements, which were considered to involve in flavonoid biosynthetic genes regulation.

*Cis*-acting elements offer important clues for the prediction of gene functions. Transcription factors cloud affect the expression levels of target genes by binding to the *cis*-acting elements of target genes during specific biological processes. To further investigate the function of the *SbPUB* genes, we predicted the *cis*-acting elements of the putative promoter regions of the *SbPUB* genes using the Plant-CARE database. As a result, 84 *cis*-acting elements were identified (**Table S3**), which were mainly associated with stress, hormones, and plant growth and development. In our study, 58 *PUB* genes had *cis*-elements related to light response, hormone response, and stress, while 53 *PUB* genes had *cis*-elements related to plant growth and development. Several diverse *cis*-acting elements were observed in the promoter region of *SbPUB*s, indicating that the *PUB* gene family of sorghum may participate in a variety of biological processes. In particular, for these *SbPUB* genes, there were many types of stress-related *cis*-elements, such as MYC, MYB, plant AP-2-like, STRE, LTR, and W box. To determine the functions of *SbPUB* genes in salt tolerance, we selected 12 interesting *cis*-acting elements, which were mainly associated with stress or stress-related hormones, for further analysis. These *cis*-acting elements were found in all *SbPUB* genes (**Figure 4**).

### Expression analysis of *SbPUB* genes under salt stress

**Figure 5:**
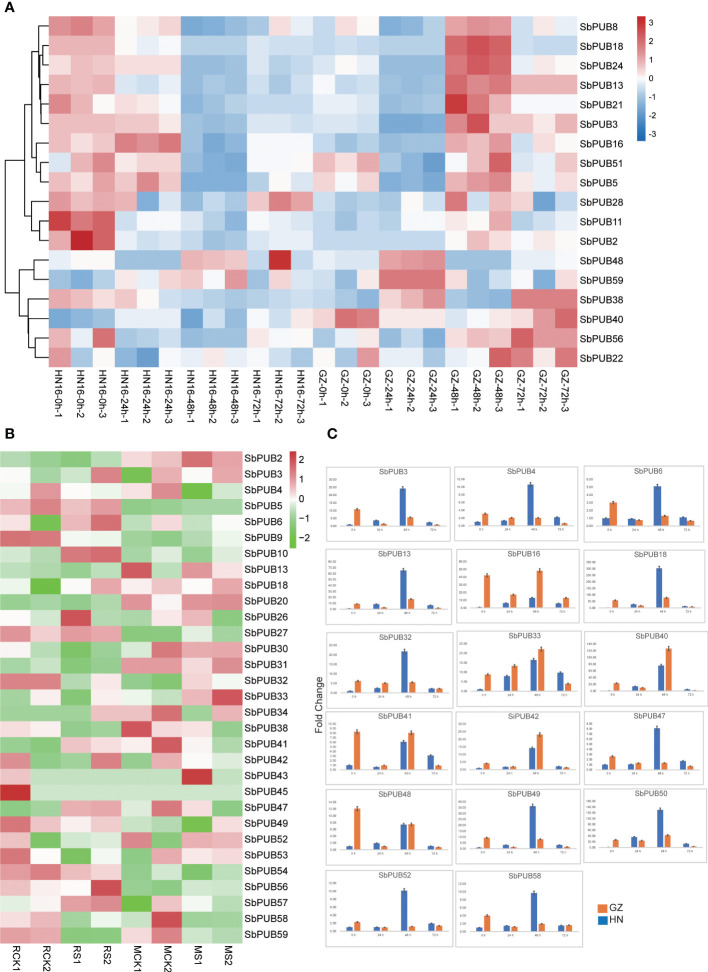
The expression analysis of the *SbPUB* gene family under salt stress. **(A)**The heatmap of the expression level of *SbPUBs* in two sorghum cultivars under different salt stresses. The expression patterns of these 18 *SbPUB* genes were based on the expression value of the proteomic data, the other *SbPUB* genes were not detected. **(B)** Expression pattern of *SbPUB* genes under salt stress in the transcriptomics data of the salt-tolerant line M-81E (M) and salt-sensitive line Roma (R). The FPKM values were obtained from RNA-seq and normalized by log_2_, and the color code represents the transformation value of log_2_(FPKM). Bright green represents low expression and dark red represents high expression. **(C)** RT-qPCR detection of DEG expression levels. Moreover, seventeen differentially expressed genes selected from the proteomic data were detected by RT-qPCR to verify the expression of the U-box gene under salt stress at 0h, 24h, 48h, and 72h.

Several studies have found that the *PUB* gene family is involved in salt tolerance. To explore the functions of the *PUB* gene family in sorghum, we detected the expression levels of *SbPUB*s based on the salt-tolerant proteomic data for two sorghum cultivars obtained in our previous study. Based on the proteomic results, 18 differentially expressed *PUB* family genes, belonging to different subfamilies, were identified (**Figure 5A**, **Table S4**). Among those genes, there were 6 genes were significantly down-regulated after 24 h, and 10 genes were significantly down-regulate after 48 h in HN16. On the contrary, these genes were significantly up-regulated after 48 h in GZ. For example, *SbPUB*13, *SbPUB*21 and *SbPUB*28 were down-regulated after 24 h in HN16 but up-regulated after 48 h in GZ. However, *SbPUB*40 and *SbPUB*48 were up-regulated after 48 h in HN16 and were not significantly differential expressed in GZ under the salt stress. *SbPUB* genes could be up-regulated under salt treated conditions in GZ, suggesting that these *SbPUB* genes play important roles under salt tolerance.

To better understand the expression of the *SbPUB* genes under salt stress, the expression levels of these genes were quantified by fragments per kilobase of transcript per million fragments mapped (FPKM) values from RNA-seq data (**Table S4**), and a heatmap was drawn according to the log2 (FPKM) values (Sun et al., 2020). The results showed that 27 *SbPUB* genes were differentially expressed in the salt-tolerant line M-81E (M) and salt-sensitive line Roma (R) (**Figure 5B**). For example, *SbPUB32* and *SbPUB38* were down-regulated in the salt-sensitive sorghum but were up-regulated in the salt-tolerant line under the salt stress. *SbPUB9* was down-regulated in salt-sensitive sorghum but the differences were minimal in the salt-tolerant line. As a result, these *PUB* genes with different expression of proteomic and transcriptional data could play a critical role in response to the salt stresses of sorghum.

Furthermore, the PPIs were visualized using these *PUB* genes with the STRING database (**Figure S1A**). The confidence score of these functional proteins is chosen to be 0.4, which means that the connections between the nodes and lines have a high degree of credibility. We found that there were extensive connections between these PUBs and other proteins. For instance, Sb03g006120.1 is a HECT-type E3 ubiquitin transferase, which plays an important role in ubiquitin-dependent protein catabolic processes (Lorenz, 2018). Both of Sb01g030340.1 and Sb01g048260.1 were ubiquitin related proteins, which showed that *PUB* has extensive links with other ubiquitin genes and plays a crucial role in the process of ubiquitin. We utilized the SWISS-MODEL tool to generate a comparative homology model of SbPUB42 with template 2c2l.1.A (SMTL ID), which had a Global Model Quality Estimate (GMQE) score of 0.71 (**Figure S1B**). Upon further analysis of the Protein Data Bank (PDB), we discovered that the Crystal structure of the CHIP U-box E3 ubiquitin ligase (PDB: 2OXQ) closely resembled the predicted structure of SbPUB42. This was evidenced by the presence of common structural features, including a central α-helix (α1), a C-terminal helix (α2), a small antiparallel β-sheet (β1 and β2), and two distinct loops (loop1 and loop2), as shown in **Figure S1C**. Notably, these same features were also observed in the GmPUB13 protein found in soybean (Lin et al., 2021). By visualizing the 3D structure of SbPUB, we can gain insights into the underlying sequence patterns, functional characteristics, and binding sites, as well as the potential interactions with other target proteins.

### qRT-PCR verification of selected *SbPUB* genes under salt stresses

To validate the expression levels of *SbPUB*s, we have selected 17 genes for qRT-PCR experiments (**Figure 5C**). The results showed that all of these *SbPUB* genes were expressed differently after salt treatment. The expression level of *SbPUB*3, *SbPUB*4, *SbPUB*6, *SbPUB*13, *SbPUB*18, *SbPUB*32, *SbPUB*47, *SbPUB*49, *SbPUB*50, *SbPUB*52, and *SbPUB*58 were highly increased during the 48 h salt exposure in the salt-sensitive HN16, and *SbPUB*16, *SbPUB*33, *SbPUB*40, *SbPUB*41, *SbPUB*42, and *SbsPUB*48 were highly increased in the salt-tolerant GZ. Moreover, *SbPUB*33 and *SbPUB*42 expressions were increased significantly within 48 h in both samples and decreased after 48 h. Similar results were found for *SbPUB*40 and *SbPUB*50, which had opposite expression patterns before 24 h but performed consistently after 24 h in two cultivars, suggesting that these *PUB* genes respond to salt treatment actively. *SbPUB16*, *SbPUB41*, and *SbPUB48* showed down-regulated before 24 h in salt-tolerant GZ, but always up-regulated before 48 h in salt-sensitive NH. Although there were some differences in expression at the initial stage (mainly before 24 h), almost all of these *SbPUB*s showed a significant reduction in expression after 48 h of salt treatment. The results of qRT-PCR were consistent with the expression trends in the proteomic and transcriptomic analyses.

## Discussion

### Genome-wide and phylogenetic analysis of *SbPUB* genes in sorghum

The U-box gene encodes a conserved U-box motif of about 70 amino acids and is a member of the ubiquitin ligase family, which regulates the ubiquitination of substrates (Yang et al., 2021). U-box genes are widely distributed in plants and participate in many biological processes, including responses to biotic and abiotic stresses (Berndsen and Wolberger, 2014; Mao et al., 2022). At present, *PUB* genes have been found to play an important role in response to salt tolerance in many plant species, such as wheat, *A. thaliana*, tomato, and soybean (Wang et al., 2016; Wang et al., 2020; Wu et al., 2020). Sorghum is one of the most important cereal crops worldwide and contains many health-promoting substances (Awika, 2017; Xu et al., 2021). However, analysis of *PUB* genes in sorghum was minimal until now. In this study, 59 genes were identified as members of the *PUB* gene family in sorghum, which was similar to tomato (62) and *A. thaliana* (64) (Heise et al., 2002; Sharma and Taganna, 2020). We found that all 59 PUB proteins contained the U-box domain, and a large number of amino acids in the SbPUB domain were highly conserved, which could play an important role in stabilizing the U-box domain (**Figure S2**). For example, arginine (R), isoleucine(I), proline (P), cysteine (C), arginine (R), threonine (T), tryptophan (W), and serine (S) were conserved; R could affect the formation of ionic bonds, and C, T, W, and S could form hydrogen bonds. Meanwhile, I may promote root development, thereby affecting the salt tolerance of plants (Yu et al., 2013).

In our study, *SbPUB* genes were classified into five groups based on both phylogenetic and structural analyses. Interestingly, motif analysis was consistent with the phylogenetic tree, and *SbPUB* genes with similar structures were found to cluster in the same groups (**Figure 2**). We found that most SbPUBs contained some other domains, such as ARM, Pkinase, WD40, USP, and TPR. We discovered that most *SbPUB* genes in Groups 2 and 4 (17 out of 23) contained an ARM repeat at the C-terminus, which could enable PPIs to regulate physiological activities in cells (Samuel et al., 2006; Tewari et al., 2010). *SbPUB13* in Group 3 contained two WD40 repeats, which might participate in transcriptional regulation and signaling (Villamil et al., 2013; Jain and Pandey, 2018). In addition, six *SbPUB* genes in Group 5 had the USP, Pkinase, and TRR domains, indicating that these proteins are involved in signal transduction *via* phosphorylation and play an important role in PPIs (Fiedler et al., 1993; Zhou et al., 2021).

Similar findings were made in other crop *PUB* studies, suggesting that the functional diversity of *PUB*s could be associated with the different motifs(Mengarelli and Zanor, 2021; Wang et al., 2021). Moreover, SbPUB, AtPUB, and OsPUB proteins were categorized into five groups based on phylogenetic analysis, which was similar to the study of rice *PUB* genes (Zeng et al., 2008). We found that the *PUB* genes of sorghum and rice were clustered into one subclade, suggesting that sorghum and rice are more closely related to each other than to *Arabidopsis*. In addition, there were 43 orthologous gene pairs between *SbPUB*s and *OsPUB*s, which was far more than that between *SbPUB*s and *AtPUB*s (**Figure 3C**). These results suggest that *SbPUB*s may have similar functions to orthologous *OsPUB*s, which will be helpful for further studies in sorghum because more *PUB* studies have been conducted in rice (Zeng et al., 2008).

### Prediction of *SbPUB* function based on *cis*-acting elements and expression analysis

Analysis of *cis*-acting elements of putative promoters suggested that the *PUB* gene family plays an important role in stress-related mechanisms, hormonal regulation, growth, and development. Previous studies have revealed that *PUB* genes could regulate salt tolerance in plants. For example, overexpression of *TaPUB15* in *Arabidopsis* and rice increased their salt tolerance, and the *PUB* gene was considered as a positive regulator of salt stress (Li et al., 2021). *TaPUB15* transgenic plants maintained a lower sodium/potassium ratio under salt stress than wild-type plants, which might be due to the deep root development and strong branching ability of the transgenic plants. Further, *TaPUB1* was found to be a positive regulator of salt stress and drought stress in wheat, as well as the positive regulator of Cd^2+^ stress tolerance (Zhang et al., 2017a; Wang et al., 2020; Zhang et al., 2021). Our results revealed that several *PUB* genes (*SbPUB2*, *SbPUB4*, *SbPUB26*, *SbPUB41*, *SbPUB52*, *SbPUB56*, *SbPUB58*) had high expressions under salt stresses in proteomic and transcriptomics data, which indicated that *SbPUB*s might have key functions in responding to salt stress in sorghum. In this study, we found several elements involved in stress-related processes, such as MBS, ARE, and MYB. In our previous study, flavonoid biological pathways were shown to have important physiological functions in the salt stress of sorghum. The MYB-binding site is an important element involved in flavonoid biosynthesis (Zhang et al., 2018). Flavonoids are a large class of polyphenol secondary metabolites, which serve a variety of functions in plant development and defense, including resistance to pathogens, insects, and environmental stress(Cominelli et al., 2005; Falcone Ferreyra et al., 2012; Liu et al., 2021). MYB-recognition elements could regulate a large number of flavonoid biosynthesis genes in many plants and play a crucial role in activating target gene promoters *in vivo* (Prouse and Campbell, 2012). Moreover, E3 ligase was found to play a vital role in regulating flavonoid biosynthesis in *Arabidopsis*, which provided a basis for exploring ubiquitin and flavonoid in sorghum (Patra et al., 2013). In this study, we discovered 12 *SbPUB* genes contained MYB-related elements, suggesting that these *SbPUB* genes might participate in resistance under salt stress and play a major role in flavonoid biosynthesis (**Figure 4**). To verify these *SbPUB* genes, we also conducted qRT-PCR experiments according the up- and downregulation of gene expression in the transcriptomic and proteomic analyses (**Figures 5A, B**). The strong agreement observed between the outcomes of qRT-PCR, transcriptome, and proteomic analyses provides compelling evidence that the SbPUB genes have a crucial function in sorghum’s response to salt stress.

## Conclusion

Environmental factors, such as drought and salinization, have severely limited agricultural productivity all over the world. Sorghum is one of the most salt-tolerant crops, and it is important to understand its salt-tolerance mechanism for crop breeding. In this study, 59 *PUB* members were identified in the sorghum genome and were unevenly distributed on 9 sorghum chromosomes. Based on phylogenetic tree analysis, *SbPUB*s were divided into five groups. The conserved motif and gene structure analyses provided strong evidence to support the classifications. *Cis*-acting element analysis indicated that *PUB* genes might participate in diverse biological processes, especially in response to salt stress. Flavonoid biosynthesis was the major pathway of sorghum salt tolerance in our previous study, while MYB-binding elements are important regulators of flavonoid biosynthetic genes in plants. We found 12 *SbPUB* genes contained the MYB-related elements, which also exhibited diverse levels of expression in the proteomic analysis. Further, qRT-PCR revealed that these genes were associated with salt stress in sorghum. These results indicate that *SbPUB* could be an important regulator in the salt tolerance of sorghum. Our study determined that MYB elements in several *PUB* genes could play a vital role in flavonoid biosynthesis under salt stress, which will aid the breeding of salt-tolerant sorghum in the future.

## Data availability statement

The original contributions presented in the study are included in the article/**Supplementary Material**. Further inquiries can be directed to the corresponding author.

## Ethics statement

The sorghum cultivars ‘Henong6’ and ‘Gaoliangzhe’ used in this study were planted and grown in Baoding, Hebei Province, China. Those two cultivars were widely planted in Hebei Province. This study did not require ethics approval and consent as wild, no endangered, or protected plant species were involved.

## Author contributions

JCu, GR, YB, YG, and JCh conceived the project. JCu, GR, and YB performed the sequences and other data analyses. YG and PY conducted the bioinformatics analyses. JCu, GR, YB, YG, and JCh prepared the manuscript. All authors contributed to the article and approved the submitted version.
