# Supplementary figures and images for: Genome-wide identification and expression analysis of the U-box E3 ubiquitin ligase gene family related to salt tolerance in sorghum (Sorghum bicolor L.)
Source: Front Plant Sci. 2023 Mar 17;14:1141617. doi: 10.3389/fpls.2023.1141617 (PMC10063820; doi:10.3389/fpls.2023.1141617)

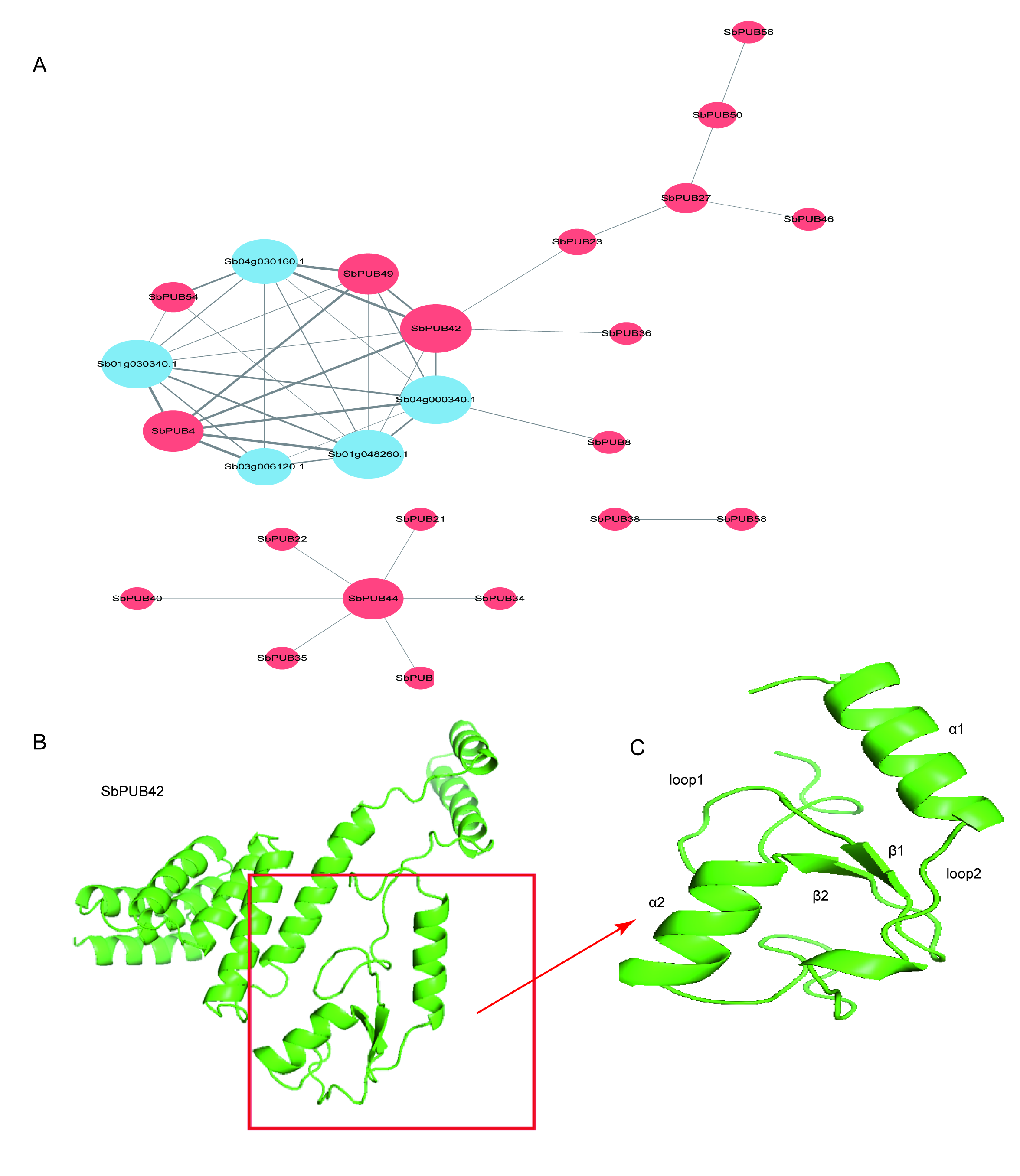

Supplement: Supplementary file 1 [file Image_1.tif]

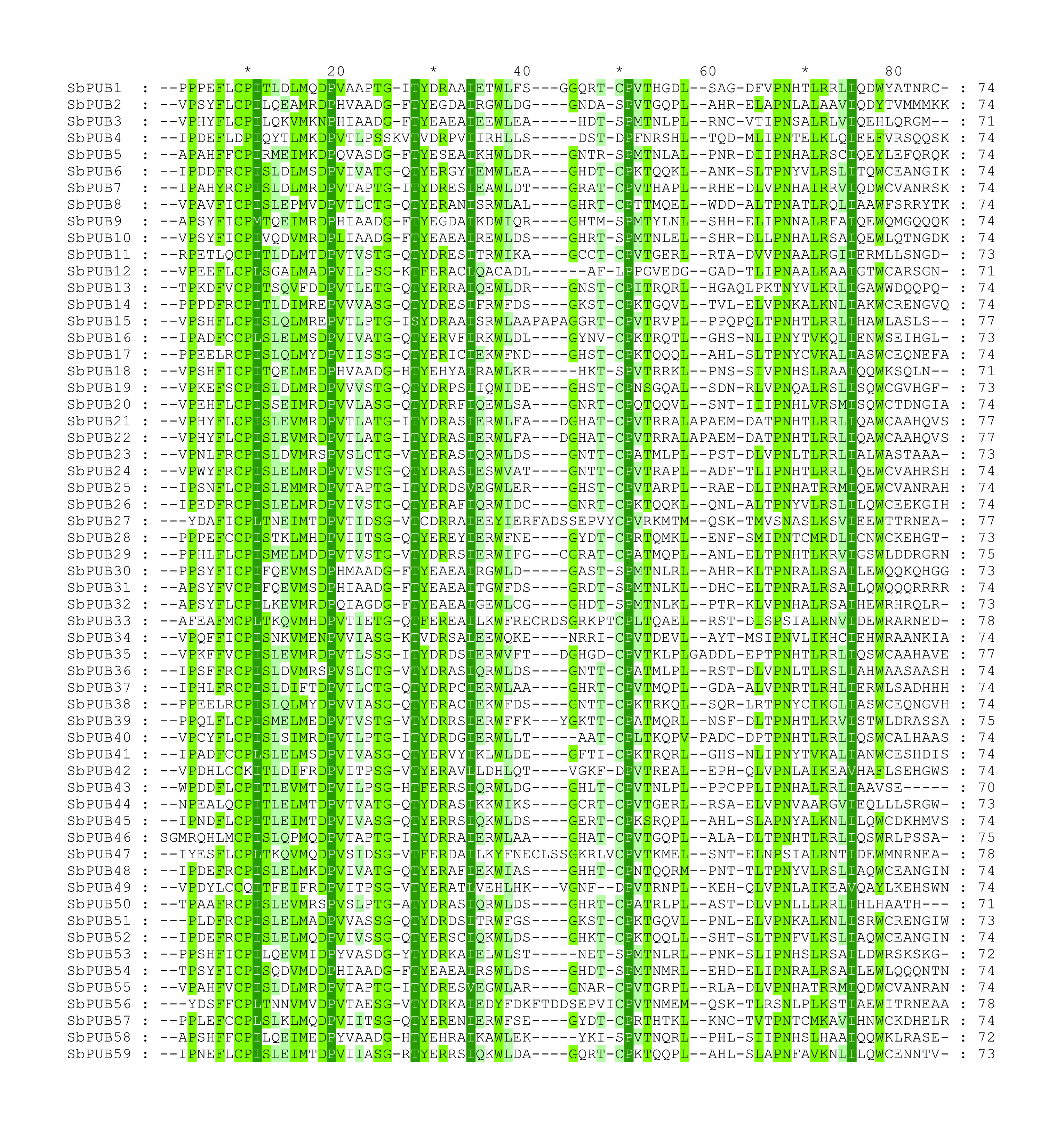

Supplement: Supplementary file 2 [file Image_2.tif]
