# Supplementary material for: Genome-wide identification and expression analysis of the U-box E3 ubiquitin ligase gene family related to salt tolerance in sorghum (Sorghum bicolor L.)
Source: Front Plant Sci. 2023 Mar 17;14:1141617. doi: 10.3389/fpls.2023.1141617 (PMC10063820; doi:10.3389/fpls.2023.1141617)
Supplement: Supplementary file 8 [file Table_6.docx]

**Genome-wide identification and expression analysis of the U-box E3 ubiquitin ligase gene family related to salt tolerance in sorghum (*Sorghum bicolor* L.)**

**Jianghui Cui ^1,2 †^, Genzeng Ren^1,2 †^, Yuzhe Bai ^1,2 †^， Yukun Gao^1,2 †^, Puyuan Yang^1,2^ , Jinhua Chang^1,2 *^**

^1^College of Agronomy, Hebei Agricultural University, Baoding, China

^2^North China Key Laboratory for Germplasm Resources of Education Ministry, Baoding, China

^†^These authors contributed equally to this work.

**^*^Correspondence:** Dr. Jinhua Chang, jhchang2006@126.com

**Number of supplementary figures/tables: 7**

**Supplementary Legends**

**Fig. S1** The protein-protein interaction (PPI) networks and the three-dimensional (3D) structure of SbPUB. (A) Interaction network of SbPUB proteins. The red circle indicated SbPUBs and the blue circle represents other sorghum genes. (B) The structure model of SbPUB42 generated by the SWISS-MODEL tool. (C)The overall structure of GmPUB13 U-box of soybean with cartoon form.

**Fig. S2** Multiple alignments of U-box domains form SbPUB proteins. The U-box domains in SbPUB proteins were predicted using MEME programs. Their sequences were aligned using ClustalX 2.1, and the alignments were edited using the GeneDoc 2.7 sequence editor.

**Table S1** Summary of the characterization of 59 *PUB* genes in sorghum.

**Table S2** List of *OsPUB*s and *AtPUB*s used in phylogenetic analysis

**Table S3** *Cis*-Acting elements analysis in the 2 kb promoters of U-box genes in sorghum.

**Table S4** Transcriptomic and proteomic data for the expression analysis.

**Table S5** Primers for RT-qPCR analysis of selected *PUB* genes in sorghum.
